# Aqua­{2-morpholino-*N*-[1-(2-pyrid­yl)ethyl­idene]ethanamine-κ^3^ *N*,*N*′,*N*′′}bis­(thio­cyanato-κ*N*)nickel(II)

**DOI:** 10.1107/S1600536810052578

**Published:** 2010-12-24

**Authors:** Nura Suleiman Gwaram, Nurul Azimah Ikmal Hisham, Hamid Khaledi, Hapipah Mohd Ali

**Affiliations:** a Department of Chemistry, University of Malaya, 50603 Kuala Lumpur, Malaysia

## Abstract

In the title compound, [Ni(NCS)_2_(C_13_H_19_N_3_O)(H_2_O)], the Ni^II^ ion is six-coordinated by the *N*,*N*′,*N*′′-tridentate Schiff base, the N atoms of two thio­cyanate ligands and one water O atom in a distorted octa­hedral geometry. Intra­molecular C—H⋯N and C—H⋯O hydrogen bonds occur. In the crystal, O—H⋯S, O—H⋯O and C—H⋯S hydrogen bonds link adjacent mol­ecules into layers parallel to the *ac* plane.

## Related literature

For the structure of the Cu(II) complex with the Schiff base and thiocyanate, see: Suleiman Gwaram *et al.* (2011 ▶). For the structures of related Ni(II) complexes, see: Chiumia *et al.* (1999 ▶); Zhao *et al.* (2008 ▶).
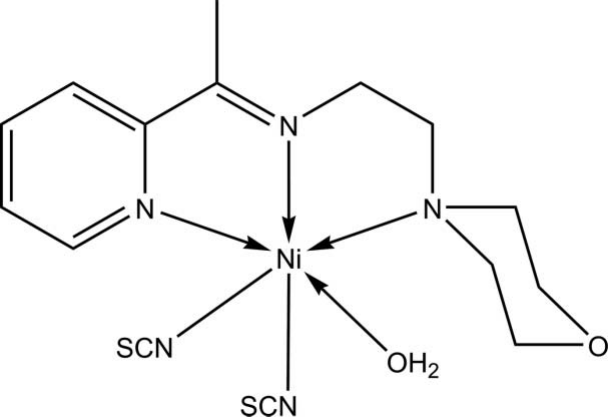


## Experimental

### 

#### Crystal data

[Ni(NCS)_2_(C_13_H_19_N_3_O)(H_2_O)]
*M* *_r_* = 426.20
Monoclinic, 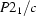

*a* = 7.1881 (1) Å
*b* = 21.9708 (3) Å
*c* = 12.1438 (2) Å
β = 91.412 (1)°
*V* = 1917.27 (5) Å^3^
*Z* = 4
Mo *K*α radiation
μ = 1.25 mm^−1^
*T* = 100 K
0.35 × 0.32 × 0.22 mm

#### Data collection

Bruker APEXII CCD diffractometer
Absorption correction: multi-scan (*SADABS*; Sheldrick, 1996 ▶) *T*_min_ = 0.669, *T*_max_ = 0.771
14240 measured reflections
3474 independent reflections
3168 reflections with *I* > 2σ(*I*)
*R*_int_ = 0.023

#### Refinement

*R*[*F* ^2^ > 2σ(*F* ^2^)] = 0.025
*wR*(*F* ^2^) = 0.058
*S* = 1.09
3474 reflections
233 parameters
4 restraints
H atoms treated by a mixture of independent and constrained refinement
Δρ_max_ = 0.45 e Å^−3^
Δρ_min_ = −0.36 e Å^−3^

### 

Data collection: *APEX2* (Bruker, 2007 ▶); cell refinement: *SAINT* (Bruker, 2007 ▶); data reduction: *SAINT*; program(s) used to solve structure: *SHELXS97* (Sheldrick, 2008 ▶); program(s) used to refine structure: *SHELXL97* (Sheldrick, 2008 ▶); molecular graphics: *X-SEED* (Barbour, 2001 ▶); software used to prepare material for publication: *SHELXL97* and *publCIF* (Westrip, 2010 ▶).

## Supplementary Material

Crystal structure: contains datablocks I, global. DOI: 10.1107/S1600536810052578/is2646sup1.cif

Structure factors: contains datablocks I. DOI: 10.1107/S1600536810052578/is2646Isup2.hkl

Additional supplementary materials:  crystallographic information; 3D view; checkCIF report

## Figures and Tables

**Table 1:** Selected bond lengths (Å)

Ni1—N1	2.1079 (16)
Ni1—N2	2.0243 (15)
Ni1—N3	2.2317 (16)
Ni1—N4	2.0270 (16)
Ni1—N5	2.0318 (16)
Ni1—O2	2.0996 (13)

**Table 2:** Hydrogen-bond geometry (Å, °)

*D*—H⋯*A*	*D*—H	H⋯*A*	*D*⋯*A*	*D*—H⋯*A*
O2—H2*A*⋯S1^i^	0.83 (1)	2.32 (1)	3.1410 (14)	168 (2)
O2—H2*B*⋯O1^ii^	0.84 (1)	1.89 (1)	2.7023 (19)	163 (2)
C2—H2⋯S1^ii^	0.95	2.84	3.769 (3)	167
C11—H11*A*⋯N4	0.99	2.53	3.409 (3)	147
C12—H12*B*⋯O2	0.99	2.40	3.100 (2)	127

Symmetry codes: (i) 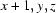; (ii) 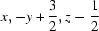.

**Fig. 1.**
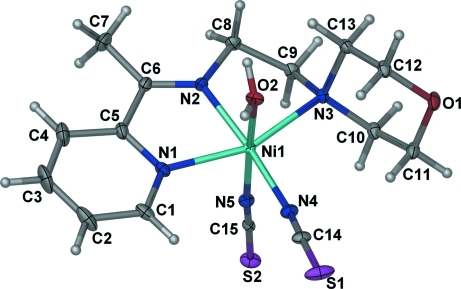
Displacement ellipsoid plot of the title compound at the 30% probability level. Hydrogen atoms are drawn as spheres of arbitrary radii.

**Fig. 2.**
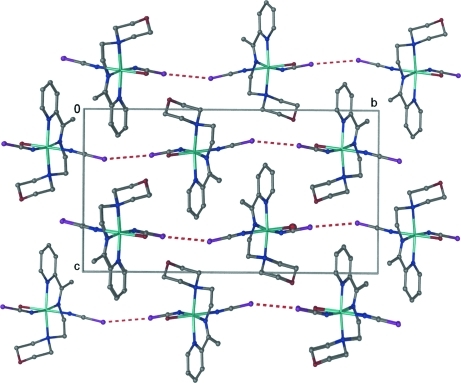
Packing view looking down the a axis, showing the intermolecular S···S interactions. H atoms have been omitted for clarity.

## supplementary crystallographic information

### Comment

The title mixed-ligand nickel(II) complex was obtained by following a similar synthetic procedure as for its analogous copper(II) complex (Suleiman Gwaram *et al.*, 2011). However, compared to the square-pyramidal environment around the Cu^II^ ion, afforded by the tridentate Schiff base and the N atoms of two SCN^-^, the Ni^II^ ion is coordinated by one additional water ligand to have an octahedral geometry. The Ni—N and Ni—O bond lengths in this complex are comparable to those in the similar structures (Chiumia *et al.*, 1999; Zhao *et al.*, 2008). In the crystal, the adjacent molecules are linked together through O—H···S, O—H···O and C—H···S hydrogen bonds into layers parallel to the *ac* plane. An S···S interaction [3.5276 (7) Å] between S1 and S2 of the symmetry related molecule at -*x*, *y* + 1/2, -*z* + 3/2, connects the layers into a three-dimensional network. Intramolecular C—H···N and C—H···O hydrogen bonding are also observed.

### Experimental

A mixture of 2-acetylpyridine (0.20 g, 1.65 mmol) and 4-(2-aminoethyl)morpholine (0.21 g, 1.65 mmol) in ethanol (20 ml) was refluxed for 2 hr followed by addition of a solution of nickel(II) acetate tetrahydrate (0.41 g, 1.65 mmol) and sodium thiocyanate (0.134 g, 1.65 mmol) in a minimum amount of water. The resulting solution was refluxed for 30 min, then left at room temperature. The crystals of the title complex were obtained in a week.

### Refinement

The C-bound H atoms were placed at calculated positions (C—H 0.95–0.99 Å) and were treated as riding on their parent C atoms. The O-bound H atoms were located in a difference Fourier map, and refined with a distance restraint of O—H 0.84 (2). For all H atoms, *U*_iso_(H) values were set to 1.2–1.5 *U*_eq_(carrier atom). An additional rigid-bond type restraint (*DELU* in *SHELXL97*) was placed on the displacement parameters of S1 and C14; S2 and C15.

### Crystal data

**Table:**

[Ni(NCS)_2_(C_13_H_19_N_3_O)(H_2_O)]	*F*(000) = 888
*M**_r_* = 426.20	*D*_x_ = 1.477 Mg m^−^^3^
Monoclinic, *P*2_1_/*c*	Mo *K*α radiation, λ = 0.71073 Å
Hall symbol: -P 2ybc	Cell parameters from 7183 reflections
*a* = 7.1881 (1) Å	θ = 2.5–30.5°
*b* = 21.9708 (3) Å	µ = 1.25 mm^−^^1^
*c* = 12.1438 (2) Å	*T* = 100 K
β = 91.412 (1)°	Block, brown
*V* = 1917.27 (5) Å^3^	0.35 × 0.32 × 0.22 mm
*Z* = 4	

### Data collection

**Table:**

Bruker APEXII CCD diffractometer	3474 independent reflections
Radiation source: fine-focus sealed tube	3168 reflections with *I* > 2σ(*I*)
graphite	*R*_int_ = 0.023
φ and ω scans	θ_max_ = 25.3°, θ_min_ = 2.5°
Absorption correction: multi-scan (*SADABS*; Sheldrick, 1996)	*h* = −8→8
*T*_min_ = 0.669, *T*_max_ = 0.771	*k* = −26→26
14240 measured reflections	*l* = −14→14

### Refinement

**Table:**

Refinement on *F*^2^	Primary atom site location: structure-invariant direct methods
Least-squares matrix: full	Secondary atom site location: difference Fourier map
*R*[*F*^2^ > 2σ(*F*^2^)] = 0.025	Hydrogen site location: inferred from neighbouring sites
*wR*(*F*^2^) = 0.058	H atoms treated by a mixture of independent and constrained refinement
*S* = 1.09	*w* = 1/[σ^2^(*F*_o_^2^) + (0.0176*P*)^2^ + 1.4584*P*] where *P* = (*F*_o_^2^ + 2*F*_c_^2^)/3
3474 reflections	(Δ/σ)_max_ = 0.002
233 parameters	Δρ_max_ = 0.45 e Å^−^^3^
4 restraints	Δρ_min_ = −0.36 e Å^−^^3^

### Special details

**Table:**

Geometry. All e.s.d.'s (except the e.s.d. in the dihedral angle between two l.s. planes) are estimated using the full covariance matrix. The cell e.s.d.'s are taken into account individually in the estimation of e.s.d.'s in distances, angles and torsion angles; correlations between e.s.d.'s in cell parameters are only used when they are defined by crystal symmetry. An approximate (isotropic) treatment of cell e.s.d.'s is used for estimating e.s.d.'s involving l.s. planes.
Refinement. Refinement of *F*^2^ against ALL reflections. The weighted *R*-factor *wR* and goodness of fit *S* are based on *F*^2^, conventional *R*-factors *R* are based on *F*, with *F* set to zero for negative *F*^2^. The threshold expression of *F*^2^ > σ(*F*^2^) is used only for calculating *R*-factors(gt) *etc*. and is not relevant to the choice of reflections for refinement. *R*-factors based on *F*^2^ are statistically about twice as large as those based on *F*, and *R*- factors based on ALL data will be even larger.

### Fractional atomic coordinates and isotropic or equivalent isotropic displacement parameters (Å^2^)

**Table:**

	*x*	*y*	*z*	*U*_iso_*/*U*_eq_	
Ni1	0.37854 (3)	0.624396 (10)	0.738472 (19)	0.01680 (7)	
S1	−0.10541 (7)	0.76958 (3)	0.71728 (7)	0.04681 (19)	
S2	0.09981 (7)	0.42948 (2)	0.80865 (5)	0.03219 (13)	
O1	0.3805 (2)	0.71032 (6)	1.05938 (12)	0.0317 (3)	
O2	0.49597 (19)	0.71103 (6)	0.71792 (12)	0.0252 (3)	
H2A	0.6074 (16)	0.7214 (11)	0.715 (2)	0.038*	
H2B	0.439 (3)	0.7350 (9)	0.6756 (17)	0.038*	
N1	0.3653 (2)	0.61959 (7)	0.56510 (13)	0.0243 (4)	
N2	0.6213 (2)	0.58250 (7)	0.70457 (13)	0.0214 (3)	
N3	0.4992 (2)	0.61675 (7)	0.90902 (13)	0.0200 (3)	
N4	0.1391 (2)	0.67219 (7)	0.75249 (15)	0.0264 (4)	
N5	0.2469 (2)	0.54344 (7)	0.75822 (14)	0.0240 (4)	
C1	0.2343 (3)	0.64192 (9)	0.49655 (18)	0.0331 (5)	
H1	0.1260	0.6594	0.5269	0.040*	
C2	0.2495 (4)	0.64071 (11)	0.3832 (2)	0.0489 (7)	
H2	0.1535	0.6567	0.3365	0.059*	
C3	0.4066 (5)	0.61587 (13)	0.3399 (2)	0.0585 (8)	
H3	0.4208	0.6147	0.2623	0.070*	
C4	0.5442 (4)	0.59253 (12)	0.40919 (19)	0.0466 (6)	
H4	0.6540	0.5754	0.3802	0.056*	
C5	0.5190 (3)	0.59464 (9)	0.52205 (17)	0.0286 (5)	
C6	0.6588 (3)	0.57188 (9)	0.60449 (17)	0.0269 (4)	
C7	0.8288 (3)	0.53922 (12)	0.5662 (2)	0.0447 (6)	
H7A	0.9150	0.5327	0.6289	0.067*	
H7B	0.8899	0.5638	0.5103	0.067*	
H7C	0.7926	0.4998	0.5345	0.067*	
C8	0.7380 (3)	0.56378 (9)	0.79863 (17)	0.0261 (4)	
H8A	0.8402	0.5934	0.8111	0.031*	
H8B	0.7932	0.5233	0.7847	0.031*	
C9	0.6158 (3)	0.56112 (9)	0.89870 (17)	0.0254 (4)	
H9A	0.5338	0.5250	0.8929	0.031*	
H9B	0.6954	0.5565	0.9659	0.031*	
C10	0.3638 (3)	0.60553 (9)	0.99758 (16)	0.0261 (4)	
H10A	0.4313	0.5907	1.0643	0.031*	
H10B	0.2751	0.5735	0.9733	0.031*	
C11	0.2574 (3)	0.66251 (9)	1.02547 (17)	0.0293 (5)	
H11A	0.1830	0.6758	0.9601	0.035*	
H11B	0.1705	0.6535	1.0854	0.035*	
C12	0.5056 (3)	0.72347 (9)	0.97228 (17)	0.0275 (4)	
H12A	0.5890	0.7573	0.9948	0.033*	
H12B	0.4335	0.7364	0.9058	0.033*	
C13	0.6201 (3)	0.66828 (9)	0.94552 (16)	0.0236 (4)	
H13A	0.7073	0.6785	0.8865	0.028*	
H13B	0.6944	0.6560	1.0115	0.028*	
C14	0.0359 (3)	0.71183 (9)	0.73737 (18)	0.0268 (4)	
C15	0.1852 (3)	0.49652 (8)	0.78008 (15)	0.0188 (4)	

### Atomic displacement parameters (Å^2^)

**Table:**

	*U*^11^	*U*^22^	*U*^33^	*U*^12^	*U*^13^	*U*^23^
Ni1	0.01653 (12)	0.01553 (12)	0.01836 (13)	0.00019 (9)	0.00068 (9)	0.00097 (9)
S1	0.0204 (3)	0.0230 (3)	0.0970 (6)	0.0010 (2)	−0.0003 (3)	0.0168 (3)
S2	0.0322 (3)	0.0209 (3)	0.0432 (3)	−0.0068 (2)	−0.0049 (2)	0.0085 (2)
O1	0.0418 (9)	0.0285 (8)	0.0253 (8)	−0.0089 (6)	0.0097 (6)	−0.0101 (6)
O2	0.0240 (7)	0.0231 (7)	0.0282 (8)	−0.0048 (6)	−0.0048 (6)	0.0097 (6)
N1	0.0283 (9)	0.0221 (8)	0.0222 (9)	−0.0049 (7)	−0.0045 (7)	0.0000 (7)
N2	0.0202 (8)	0.0219 (8)	0.0220 (9)	0.0012 (6)	0.0013 (7)	−0.0016 (7)
N3	0.0244 (8)	0.0183 (8)	0.0174 (8)	−0.0001 (6)	0.0013 (6)	0.0001 (6)
N4	0.0210 (8)	0.0222 (8)	0.0361 (10)	0.0012 (7)	0.0013 (7)	0.0014 (7)
N5	0.0227 (8)	0.0195 (8)	0.0298 (9)	−0.0001 (7)	0.0009 (7)	−0.0015 (7)
C1	0.0408 (12)	0.0251 (10)	0.0325 (12)	−0.0051 (9)	−0.0150 (10)	0.0002 (9)
C2	0.0769 (19)	0.0364 (13)	0.0320 (14)	−0.0005 (13)	−0.0256 (13)	−0.0015 (11)
C3	0.098 (2)	0.0587 (17)	0.0186 (12)	−0.0007 (16)	−0.0089 (14)	−0.0053 (12)
C4	0.0644 (17)	0.0520 (15)	0.0236 (12)	−0.0010 (13)	0.0053 (11)	−0.0094 (11)
C5	0.0353 (11)	0.0300 (11)	0.0205 (11)	−0.0069 (9)	0.0023 (9)	−0.0044 (9)
C6	0.0238 (10)	0.0304 (11)	0.0267 (11)	−0.0021 (8)	0.0051 (8)	−0.0059 (9)
C7	0.0322 (12)	0.0589 (16)	0.0437 (15)	0.0059 (11)	0.0123 (11)	−0.0165 (12)
C8	0.0226 (10)	0.0261 (10)	0.0293 (11)	0.0067 (8)	−0.0029 (8)	−0.0007 (9)
C9	0.0309 (11)	0.0204 (10)	0.0247 (11)	0.0060 (8)	−0.0048 (9)	0.0027 (8)
C10	0.0356 (11)	0.0230 (10)	0.0197 (10)	−0.0058 (8)	0.0039 (9)	0.0014 (8)
C11	0.0346 (11)	0.0289 (11)	0.0250 (11)	−0.0077 (9)	0.0099 (9)	−0.0057 (9)
C12	0.0368 (11)	0.0235 (10)	0.0224 (11)	−0.0071 (9)	0.0040 (9)	−0.0034 (8)
C13	0.0280 (10)	0.0244 (10)	0.0184 (10)	−0.0052 (8)	−0.0022 (8)	0.0002 (8)
C14	0.0186 (9)	0.0207 (9)	0.0410 (12)	−0.0037 (7)	0.0000 (9)	0.0030 (9)
C15	0.0192 (9)	0.0185 (8)	0.0184 (9)	0.0004 (7)	−0.0026 (7)	−0.0015 (7)

### Geometric parameters (Å, °)

**Table:**

Ni1—N1	2.1079 (16)	C3—C4	1.382 (4)
Ni1—N2	2.0243 (15)	C3—H3	0.9500
Ni1—N3	2.2317 (16)	C4—C5	1.388 (3)
Ni1—N4	2.0270 (16)	C4—H4	0.9500
Ni1—N5	2.0318 (16)	C5—C6	1.487 (3)
Ni1—O2	2.0996 (13)	C6—C7	1.501 (3)
S1—C14	1.640 (2)	C7—H7A	0.9800
S2—C15	1.6362 (19)	C7—H7B	0.9800
O1—C11	1.428 (2)	C7—H7C	0.9800
O1—C12	1.434 (2)	C8—C9	1.518 (3)
O2—H2A	0.834 (10)	C8—H8A	0.9900
O2—H2B	0.835 (10)	C8—H8B	0.9900
N1—C1	1.335 (3)	C9—H9A	0.9900
N1—C5	1.350 (3)	C9—H9B	0.9900
N2—C6	1.273 (3)	C10—C11	1.510 (3)
N2—C8	1.459 (2)	C10—H10A	0.9900
N3—C13	1.488 (2)	C10—H10B	0.9900
N3—C10	1.488 (2)	C11—H11A	0.9900
N3—C9	1.489 (2)	C11—H11B	0.9900
N4—C14	1.156 (3)	C12—C13	1.505 (3)
N5—C15	1.156 (2)	C12—H12A	0.9900
C1—C2	1.384 (3)	C12—H12B	0.9900
C1—H1	0.9500	C13—H13A	0.9900
C2—C3	1.371 (4)	C13—H13B	0.9900
C2—H2	0.9500		
			
N2—Ni1—N4	172.20 (7)	N2—C6—C5	115.19 (18)
N2—Ni1—N5	91.85 (6)	N2—C6—C7	125.2 (2)
N4—Ni1—N5	92.56 (6)	C5—C6—C7	119.60 (19)
N2—Ni1—O2	92.11 (6)	C6—C7—H7A	109.5
N4—Ni1—O2	83.40 (6)	C6—C7—H7B	109.5
N5—Ni1—O2	175.94 (6)	H7A—C7—H7B	109.5
N2—Ni1—N1	77.99 (6)	C6—C7—H7C	109.5
N4—Ni1—N1	95.30 (7)	H7A—C7—H7C	109.5
N5—Ni1—N1	93.69 (6)	H7B—C7—H7C	109.5
O2—Ni1—N1	86.28 (6)	N2—C8—C9	107.73 (15)
N2—Ni1—N3	80.64 (6)	N2—C8—H8A	110.2
N4—Ni1—N3	105.80 (6)	C9—C8—H8A	110.2
N5—Ni1—N3	89.77 (6)	N2—C8—H8B	110.2
O2—Ni1—N3	91.75 (5)	C9—C8—H8B	110.2
N1—Ni1—N3	158.45 (6)	H8A—C8—H8B	108.5
C11—O1—C12	109.31 (14)	N3—C9—C8	111.96 (15)
Ni1—O2—H2A	129.9 (17)	N3—C9—H9A	109.2
Ni1—O2—H2B	117.1 (17)	C8—C9—H9A	109.2
H2A—O2—H2B	105 (2)	N3—C9—H9B	109.2
C1—N1—C5	118.65 (19)	C8—C9—H9B	109.2
C1—N1—Ni1	128.17 (15)	H9A—C9—H9B	107.9
C5—N1—Ni1	112.94 (13)	N3—C10—C11	111.62 (16)
C6—N2—C8	124.37 (17)	N3—C10—H10A	109.3
C6—N2—Ni1	118.78 (14)	C11—C10—H10A	109.3
C8—N2—Ni1	116.78 (12)	N3—C10—H10B	109.3
C13—N3—C10	107.34 (14)	C11—C10—H10B	109.3
C13—N3—C9	108.88 (15)	H10A—C10—H10B	108.0
C10—N3—C9	107.71 (14)	O1—C11—C10	111.17 (17)
C13—N3—Ni1	115.45 (11)	O1—C11—H11A	109.4
C10—N3—Ni1	115.92 (12)	C10—C11—H11A	109.4
C9—N3—Ni1	100.97 (11)	O1—C11—H11B	109.4
C14—N4—Ni1	156.93 (16)	C10—C11—H11B	109.4
C15—N5—Ni1	172.08 (16)	H11A—C11—H11B	108.0
N1—C1—C2	122.8 (2)	O1—C12—C13	110.66 (16)
N1—C1—H1	118.6	O1—C12—H12A	109.5
C2—C1—H1	118.6	C13—C12—H12A	109.5
C3—C2—C1	118.4 (2)	O1—C12—H12B	109.5
C3—C2—H2	120.8	C13—C12—H12B	109.5
C1—C2—H2	120.8	H12A—C12—H12B	108.1
C2—C3—C4	119.9 (2)	N3—C13—C12	111.02 (16)
C2—C3—H3	120.1	N3—C13—H13A	109.4
C4—C3—H3	120.1	C12—C13—H13A	109.4
C3—C4—C5	118.7 (2)	N3—C13—H13B	109.4
C3—C4—H4	120.6	C12—C13—H13B	109.4
C5—C4—H4	120.6	H13A—C13—H13B	108.0
N1—C5—C4	121.6 (2)	N4—C14—S1	178.19 (19)
N1—C5—C6	114.94 (17)	N5—C15—S2	178.78 (18)
C4—C5—C6	123.5 (2)		

### Hydrogen-bond geometry (Å, °)

**Table:**

*D*—H···*A*	*D*—H	H···*A*	*D*···*A*	*D*—H···*A*
O2—H2A···S1^i^	0.83 (1)	2.32 (1)	3.1410 (14)	168 (2)
O2—H2B···O1^ii^	0.84 (1)	1.89 (1)	2.7023 (19)	163 (2)
C2—H2···S1^ii^	0.95	2.84	3.769 (3)	167.
C11—H11A···N4	0.99	2.53	3.409 (3)	147
C12—H12B···O2	0.99	2.40	3.100 (2)	127

Symmetry codes: (i) *x*+1, *y*, *z*; (ii) *x*, −*y*+3/2, *z*−1/2.
